# Behaviour and Physiology: The Thermal Strategy of Leatherback Turtles

**DOI:** 10.1371/journal.pone.0013925

**Published:** 2010-11-10

**Authors:** Brian L. Bostrom, T. Todd Jones, Mervin Hastings, David R. Jones

**Affiliations:** 1 Department of Zoology, University of British Columbia, Vancouver, British Columbia, Canada; 2 Joint Institute for Marine and Atmospheric Research, University of Hawaii at Manoa, National Oceanic and Atmospheric Administration, Kewalo Research Facility, Honolulu, Hawaii, United States of America; 3 Conservation and Fisheries Department, Ministry of Natural Resources and Labour, Government of the British Virgin Islands, Road Town, Tortola, British Virgin Islands; Roehampton University, United Kingdom

## Abstract

**Background:**

Adult leatherback turtles (*Dermochelys coriacea*) exhibit thermal gradients between their bodies and the environment of ≥8°C in sub-polar waters and ≤4°C in the tropics. There has been no direct evidence for thermoregulation in leatherbacks although modelling and morphological studies have given an indication of how thermoregulation may be achieved.

**Methodology/Principal Findings:**

We show for the first time that leatherbacks are indeed capable of thermoregulation from studies on juvenile leatherbacks of 16 and 37 kg. In cold water (< 25°C), flipper stroke frequency increased, heat loss through the plastron, carapace and flippers was minimized, and a positive thermal gradient of up to 2.3°C was maintained between body and environment. In warm water (25 – 31°C), turtles were inactive and heat loss through their plastron, carapace and flippers increased. The thermal gradient was minimized (0.5°C). Using a scaling model, we estimate that a 300 kg adult leatherback is able to maintain a maximum thermal gradient of 18.2°C in cold sub-polar waters.

**Conclusions/Significance:**

In juvenile leatherbacks, heat gain is controlled behaviourally by increasing activity while heat flux is regulated physiologically, presumably by regulation of blood flow distribution. Hence, harnessing physiology and behaviour allows leatherbacks to keep warm while foraging in cold sub-polar waters and to prevent overheating in a tropical environment.

## Introduction

Body temperature (*T*_B_) has a pronounced effect on all metabolic processes and, for many ectotherms, maintaining *T*_B_ within a certain temperature range can be advantageous both behaviourally and metabolically. To regulate *T*_B_ ectotherms employ a thermal strategy that has both physiological and behavioural components which alter the rate of heat loss or gain [1]. However, for the majority of ectotherms metabolic heat is not integral to their thermal biology. Due to the lack of an internal heat source most fish, amphibians and reptiles maintain optimum physiological performance only across a narrow range of ambient temperatures [2]–[4]. Nevertheless some animals, such as the leatherback sea turtle, can maintain *T*_B_ near optimum over a large range of ambient temperatures which expands their thermal niche and greatly increases their global range [5].

Leatherback sea turtles are large oceanic pelagic reptiles that nest in the tropics where the water temperature (*T*_W_) can be as high as 30°C [6] and spend extended periods of time foraging in cold northern waters that approach 0°C [5]. No other known reptile inhabits such a large ambient temperature range. Leatherbacks swimming in tropical waters have body temperatures (*T*_B_) 1.2 – 4.3°C above ambient *T*_W_ [6]. In contrast, leatherbacks captured in foraging grounds off of Nova Scotia (Canada) typically maintained *T*_B_ of 24.3°C in surface water of 16.1°C for a thermal gradient (*T*_B_–*T*_W_) of at least 8.2°C [7]. Currently, the precise mechanisms involved in the leatherbacks' thermoregulatory ability are poorly understood.

Leatherbacks are thought to draw on a suite of physiological and behavioral adaptations to regulate their rate of heat loss and gain. Based on biophysical modeling it was concluded that large body size and use of peripheral tissues as insulation coupled with the ability to control heat flux via circulatory adjustments would allow leatherbacks to regulate *T*_B_ in both warm and cold waters [8]. Furthermore, leatherbacks are thought to possess counter-current heat exchangers in both the anterior and posterior flippers [9], have thick layers of adipose tissue surrounding cranial structures including the esophagus to prevent heat loss from the animal's head [10] and have peripheral layers of fat which include deposits of brown adipose tissue [11]. However, the *T*_B_ – *T*_W_ a leatherback maintains not only depends on the rate of heat loss, but also on the rate of heat production. Recently, the importance of behavioral adjustments (i.e. swimming activity) as a further thermoregulatory mechanism to maintain preferred *T*_B_ – *T*_W_ in different thermal environments was analyzed [12]. Taken together, results of previous studies have suggested integrated roles of large body size and physiological and behavioral adjustments in leatherback thermoregulation.

While different components of leatherback thermal biology have been measured and/or modelled in several studies, an holistic approach to quantifying the collective leatherback thermoregulatory response is lacking. In this study, we report the first empirical observations of the physiological and behavioral responses of leatherbacks to controlled variations in thermal environment. We apply our results to thermoregulation of leatherbacks, from juveniles to adults, in their natural environment, from tropical to polar seas.

## Results

### 1. Thermal gradient

**Figure 1:**
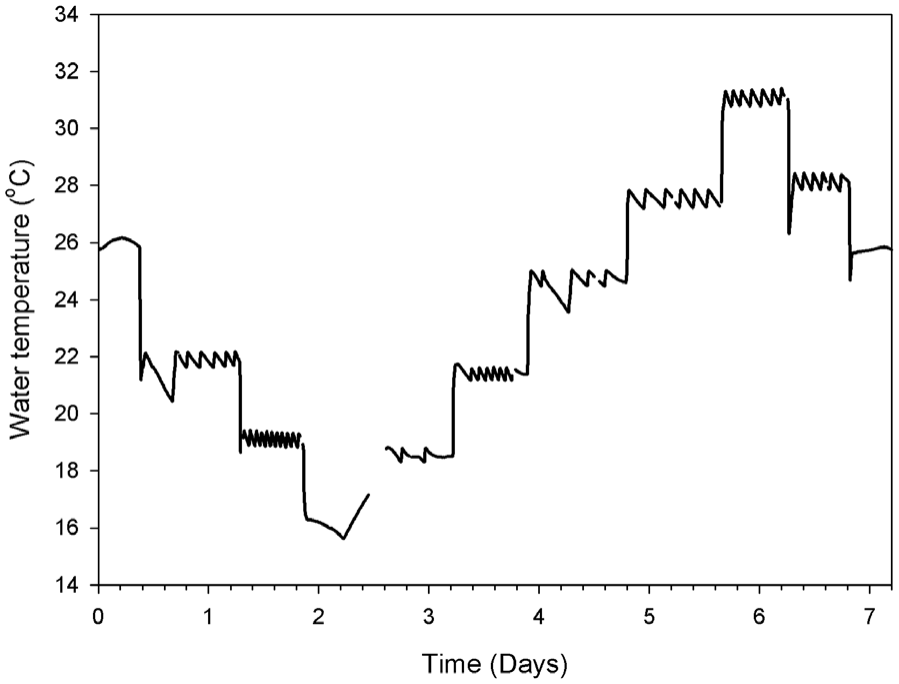
The leatherbacks were exposed to stepwise changes in water temperature. The complete water temperature profile for the experiment performed on the 37 kg leatherback.

As tank water was cooled stepwise from the acclimation temperature of 25°C (for *T*_W_ change protocol see Figure 1) both study animals maintained a progressively larger thermal gradient between their body and the water (Table 1 and 2). At *T*_W_ 25°C the 37 kg turtle maintained a *T*_B_ – *T*_W_ of 0.9°C which increased to 2.3°C in *T*_W_ of 16°C (Table 1). The 16 kg animal started off with a gradient of 1.5°C which increased to 2.0°C in the coldest water (Table 2). The 37 kg animal's thermal gradient decreased to 0.5°C and the 16 kg turtle's gradient was 0.8°C in water at 31°C. Overall a trend was found in both leatherbacks where cooler water led to the maintenance of larger thermal gradients, but *T*_B_ – *T*_W_ gradients in both animals were greater when water temperature was reduced to a given *T*_W_ compared with being raised to the same *T*_W_. Additionally, the larger turtle displayed a larger variation in thermal gradients (0.5 to 2.3°C) when compared with the smaller turtle (0.8 to 2.0°C) over the *T*_W_ range tested.

**Table 1:** A leatherback's rate of heat production and heat loss depend on water temperature.

Water temperature (°C)	Thermal Gradient *T*_B_- *T*_W_ (°C)	Flipper stroke frequency (SPM)	Flipper Heat Loss Rate (W m^2^)	Plastron Heat Loss Rate (W m^2^)	Thermal Admittance (W m^2^ K^−1^)	Total Heat Loss (W kg^−1^)	Flipper Heat Loss(% total heat loss)
**25**	0.9	3	6.5	12.8	14.4	0.24	16
**22**	1.4	21	5.8	16.0	11.4	0.29	12
**19**	1.6	25	11.0	19.5	12.0	0.38	18
**16**	2.3	29	4.6	26.2	11.5	0.44	6
**19**	1.0	19	2.8	10.9	10.4	0.19	9
**22**	0.7	5	5.4	10.7	14.4	0.20	16
**25**	1.1	3	5.7	10.9	10.4	0.21	17
**28**	0.7	3	16.5	13.8	20.6	0.32	32
**31**	0.5	3	14.6	13.5	29.5	0.30	29
**28**	0.5	8	14.3	14.0	26.9	0.31	28
**25**	0.9	6	2.4	9.4	10.4	0.16	9

Recorded and calculated values at each water temperature for the 37 kg leatherback.

**Table 2:** Recorded and calculated values at each water temperature for the 16 kg leatherback.

Water temperature (°C)	Thermal Gradient*T*_B_- *T*_W_ (°C)	Flipper stroke frequency (SPM)	Flipper Heat Loss Rate (W m^2^)	Plastron Heat Loss Rate (W m^2^)	Thermal Admittance (W m^2^ K^−1^)	Total Heat Loss (W kg^−1^)	Flipper Heat Loss(% total heat loss)
**25**	1.5	20	6.7	14.5	9.9	0.35	14
**22**	1.4	22	4.0	12.3	8.7	0.28	10
**19**	1.6	30	3.1	-	-	-	-
**16**	2.0	36	3.3	15.7	7.9	0.35	7
**19**	1.0	29	4.0	8.2	8.6	0.20	15
**22**	0.8	13	3.0	8.9	11.1	0.21	11
**25**	1.2	2	6.2	9.1	7.9	0.23	20
**28**	1.0	2	8.6	8.8	8.4	0.24	26
**31**	0.8	2	7.4	-	-	-	-
**28**	-	3	-	11.1	-	-	-
**25**	-	9	-	-	-	-	-

### 2. Swimming activity

In tank water 22°C and higher the 37 kg leatherback was nearly inactive swimming at a flipper stroke frequency ranging between 2 and 8 strokes per minute, SPM (Table 1). The 16 kg leatherback on the other hand became nearly inactive in water that was any warmer 22°C (Table 2). Stroke frequency greatly increased in both leatherbacks after a drop in temperature from 22°C. After each reduction in *T*_W_ the animals stroke rate increased and the rate was maintained over the entire time the turtle was in that *T*_W_. In the coldest water (16°C) the 37 and 16 kg leatherbacks maintained their highest average activity rates at 29 and 36 SPM, respectively.

### 3. Heat loss

When *T*_W_ was 25°C or less the 37 kg leatherback lost <7 W m^−2^, through the front flippers except when cooled to 19°C when heat flux from the front flippers, *Q*_F_, rose to 11.0 W m^−2^ (Table 1). When warmed to 28°C heat flux increased substantially to 16 W m^2^. A similarly high *Q*_F_ was maintained until the animal was re-cooled to 25°C. The 16 kg leatherback lost <4 W m^−2^ through its flippers when tank water was 22°C and below (Table 2). When at 25°C heat flux increased to 6.2 W m^−2^ and at 28°C increased a further 39% to 8.6 W m^−2^. At 31°C *Q*_F_ of the smaller turtle dropped slightly to 7.4 W m^−2^.

The 37 kg leatherback lost 12.8 W m^−2^ through the plastron at the acclimation temperature and this value steadily rose to 26.2 W m^−2^ in the coldest water (Table 1). Upon re-warming to 19°C heat flux through the plastron, *Q*_P_, dropped to 10.9 W m^−2^. This heat flux was maintained until 28°C when it increased slightly to 13.8 W m^−2^ remaining stable until returned to 25°C when plastron heat flux dropped to 9.4 W m^−2^. The 16 kg leatherback followed a similar trend with heat flux highest in 16°C water (15.7 W m^−2^) and a steady heat flux around 9 W m^−2^ from 19 through 28°C. The rate of heat flux from the carapace of the 16 kg leatherback was nearly the same as *Q*_P_ at each *T*_W_ tested.

When the 37 kg leatherback was in *T*_W_ ≤25°C , thermal admittance (see Materials & Methods for explanation of thermal admittance) was between 10 and 14 W m^−2^°C^−1^ (Table 1). When the tank water was warmed to 28°C, the thermal admittance nearly doubled to 20.6 W m^2^ K^−1^ and in 31°C water reached 29.5 W m^2^ K^−1^, nearly triple the cold water value. On cooling the animal to its acclimation temperature of 25°C thermal admittance fell to 10.4 W m^2^ K^−1^. The 16 kg turtle had a constant value for thermal admittance around 9 W m^2^ K^−1^ in all water temperatures tested. There was insufficient data, unfortunately, to calculate the thermal admittance in 31°C *T*_W_ for the smaller turtle.

### 4. Surface area

The total surface area of the plastron and carapace, *A*_B_, was 0.64 and 0.42 m^2^ for the 42.0 and 22.3 kg leatherback carcasses, respectively. The area of the front and rear flippers, *A*_F_, was 0.25 and 0.15 m^2^ for the large and small turtle, respectively. Total body area (m^2^), was fitted to a power function and found to scale with *M*_B_ (kg) as *A*_B_ = 0.049 *M*_B_^0.69^ and the area of all four flippers scaled as *A*_F_ = 0.014 *M*_B_^0.77^. Using these equations, at the time of the experiments, *A*_B_ for the smaller 16 kg turtle was 0.33 m^2^ and the 37 kg turtle was 0.59 m^2^. *A*_F_ was 0.12 m^2^ for the small and 0.22 m^2^ for the larger turtle.

### 5. Total heat loss

At 25°C the 37 kg leatherback lost 0.24 W kg^−1^ from the body and flippers, and this total heat loss, *q*_T_, rose steadily to 0.44 W kg^−1^ in the coldest water (Table 1). When warmed to 19°C, *q*_T_ dropped to 0.19 W kg^−1^ and this value varied little until 28°C when it rose to 0.32 W kg^−1^ (see Figure 1 for *T_W_* profile). This *q*_T_ was held steady until the animal was again cooled to 25°C. The smaller turtle at 25°C had a *q*_T_ of 0.35 W kg^−1^ which fell to 0.28 W kg^−1^ in 22°C water but increased to 0.35 W kg^−1^ in 16°C water. Upon re-warming to 19°C, *q*_T_ dropped to 0.20 W kg^−1^ and then slowly increased to 0.24 W kg^−1^ at 28°C.

### 6. Fraction of heat loss through the body and carapace

In the coldest water (16°C) both turtles lost about 7% of *q*_T_ through their flippers with the remaining 93% being lost through the body (ie. plastron and carapace, Table 1 and 2). The proportion of heat lost from the flippers increased as *T*_W_ rose in both turtles. In 28 and 31°C *T*_W_ around 30% of *q*_T_ was lost from the flippers compared with 70% from the plastron and carapace.

## Discussion

There has been considerable speculation that leatherbacks are endothermic and able to thermoregulate based upon the fact that their global range spans from cold northern foraging grounds to tropical nesting beaches. In the coldest water we tested (16°C) the 16 and 37 kg leatherback maintained a thermal gradient of 2.0 and 2.3°C, respectively, while in the warmest water (31°C) the thermal gradient was reduced to 0.5 and 0.8°C (Table 1 and 2). Therefore, we have shown for the first time, using juveniles in a controlled temperature environment, that leatherbacks possess the ability to hold and regulate their thermal gradient. Furthermore, since the heat energy to hold these thermal gradients is metabolically derived the animals are, by definition, endothermic.

### 1. Physiological and behavioural responses to warm and cold water

**Figure 2:**
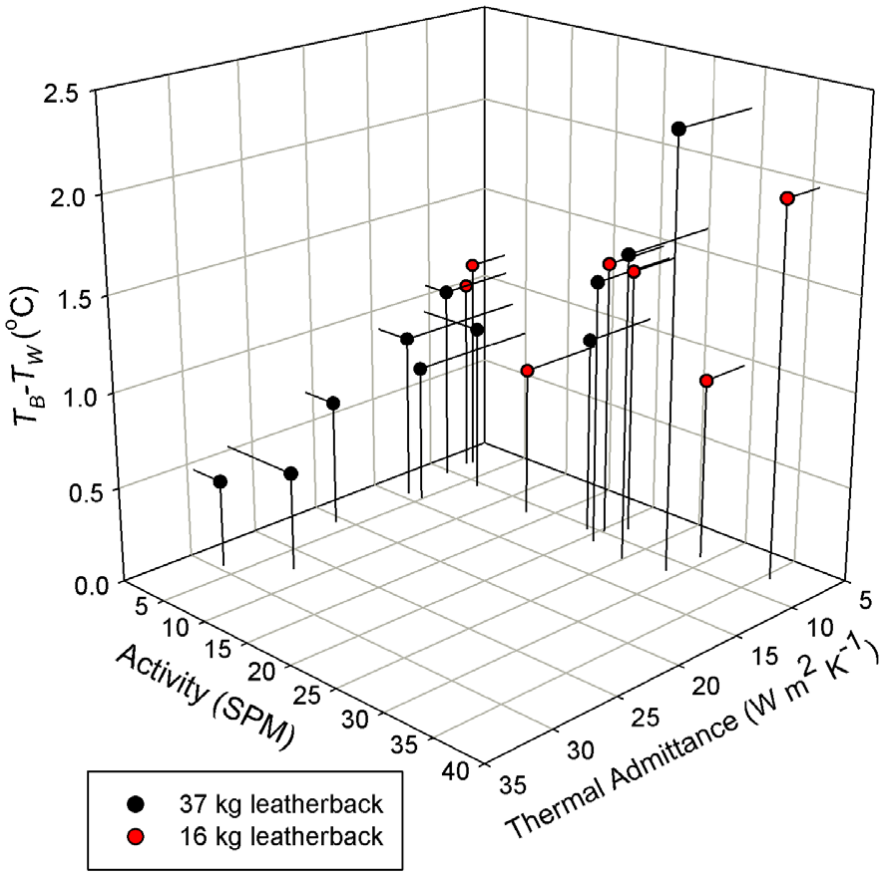
Leatherbacks thermoregulate by controlling both heat loss and heat gain. A 3D image showing how activity (heat production) and thermal admittance (heat loss) affect the thermal gradient (*T_B_-T_W_*) held by juvenile leatherbacks. In cold water heat loss was minimized and activity was proportional to the thermal gradient held. In warm water activity was very low and thermal gradient was due to varying heat loss.

In water colder than their acclimation temperature (*T*_W_ <25°C) the flipper stroke frequency of both leatherbacks increased as *T*_W_ decreased (Table 1 and 2). Since each flipper stroke causes water movement the activity rate is proportional to the power output by the turtle. Due to the inefficiency of metabolic processes, as power output increases heat production must increase as well. Therefore as *T*_W_ got colder endogenous heat production increased. In the coldest water flipper stroke frequency was highest and the largest thermal gradient was held. *T*_B_ – *T*_W_ during cooling was different from that during re-warming and a similar hysteresis was seen in the relation between flipper stroke frequency and *T*_W_. Further, *T*_B_ – *T*_W_ varied in association with activity for *T*_B_ – *T*_W_'s between 1 and 2.3°C. Since both leatherbacks maintained a stable thermal admittance during those trials the *T*_B_ – *T*_W_'s were due largely to activity (Figure 2). The potential to use behavioral control of activity to regulate heat production and therefore *T*_B_ [12] has been confirmed in our study. Eckert (2002) found leatherbacks swim continuously which shows their potential to maintain high activity rates over very long periods of time [13]. Behavioral control of heat production has not been shown to be an integral thermoregulatory mechanism in any other reptile but it is probable that it may be used in other endothermic species such as lamnid sharks, tunas and billfish, especially when exposed to very cold waters. Behavioural control of heat production contrasts with other endotherms in which metabolic heat production is largely controlled autonomically.

Even though the surface area of the front and rear flippers combined represents 27% of the total surface area of each turtle we found that only 6–7% of total heat loss, *q*_T_, came from the flippers in the coldest water (Table 1 and 2). Despite not having the ability to completely halt heat flux, the flippers are responsible for only a small fraction of total heat lost. In warm water heat flux increases substantially from the flippers to a rate nearly 3 times that in cold water and accounts for 30% of *q*_T_. These observations suggest an effective control over flipper heat flux, either by utilization of counter-current heat exchangers or, at its simplest, a reduction in total blood flow to the flippers in cold water. A dense, intertwining network of arteries and veins in both the anterior and posterior flippers have been histologically described and provide anatomical evidence for heat exchangers [9]. Heat exchangers have evolved independently in lamnid sharks and scombrids, underscoring the primary importance of heat retention in maintenance of elevated body temperatures in each lineage [14], [15]. No evidence of heat exchangers in green and loggerhead sea turtles has been reported, but both species have been shown to greatly reduce blood flow to their flippers when exposed to *T*_W_ lower than their acclimation temperatures [16]. In addition, thermal effects on the whole circulation could have an important influence on heat flux. Nonetheless, despite the lack of clarity on the precise mechanism controlling heat loss from the flippers there is no doubt that the flippers are an important part of a leatherbacks thermal arsenal.

In contrast to the flippers, leatherbacks lost a substantial amount of heat energy through the plastron at all water temperatures (Table 1 and 2). Since the body was always warmer than tank water, heat was conducted from the core of the animal to the water. If heat passed to the water solely by conduction, heat flux should be directly proportional to the thermal gradient across the plastron (Eq. 3) with any deviation reflecting physiological changes such as a variation in blood flow. Therefore, the thermal admittance, the heat flux for a given thermal gradient, should be an accurate index of physiological blood flow changes a leatherback makes that affect *Q*_P_. In *T*_W_ ≤25°C the 37 kg leatherback maintained a stable thermal admittance of around 12 W m^−2^ K^−1^ (Table 1) and at all temperatures tested the 16 kg leatherback also maintained a constant thermal admittance, although slightly lower at 9 W m^−2^ K^−1^ (Table 2). When in colder water, than the acclimation temperature, little heat energy was lost through their flippers so physiologically their thermal insulation was at a maximum due to little blood flow to the skin surface. In contrast, in water above the acclimation temperature, thermal admittance nearly tripled so more heat was lost convectively and blood flow to the skin must have increased. As well, *T*_B_ – *T*_W_ correlated closely with thermal admittance when *T*_B_ – *T*_W_ was below 1°C (Figure 2). Since activity was minimized the physiological changes affected the *T*_B_ – *T*_W_ confirming the suggestion that leatherbacks could control *T*_B_ through blood flow [8].

Interestingly, the thermal admittance of the large leatherback was around 30% greater than that of the small turtle at 16°C. Therefore at a given gradient between *T_B_* and *T_W_* the larger turtle lost around 30% more heat per unit area from its plastron than the small turtle, regardless of the gradient between body and water. The smaller turtle had a thinner plastron and therefore we expected it to have a thinner layer of insulation and consequently a higher heat flux for a given *T*_B_ – *T*_W_. Since thermal admittance is equal to thermal conductivity, *k*, divided by insulation thickness, *L*, (see Methods) either the smaller turtle has a less thermally conductive insulation layer, lower *k*, or the insulating layer is thicker than in the larger turtle. It is possible that the thermal gradient we measured between the body and tank water did not truly represent the thermal gradient directly across the plastron. *T*_B_ was measured as gastrointestinal tract temperature and although core body temperature is generally modelled as being homogenous due to blood flow [8], [12], this may not always be the case. Heat lost through the plastron could cause a temperature profile inside the turtle where the gastrointestinal tract of the animal is at *T*_B_ and peripheral tissues near the plastron are closer to *T_W_* because of inadequate perfusion and the temperature approaches *T*_W_ closer to the plastron. In essence, since heat around the gastrointestinal tract is now passing through these cooler peripheral tissues largely by conduction, the tissues would have acted as insulation, increasing *L,* and leading to a lower thermal admittance in the smaller turtle. In cold water, the entire body cavity of the larger turtle is likely more homogenous in temperature than the smaller turtle because the plastron and carapace are thicker, will provide better insulation, and there is a larger volume of blood circulating endogenously produced heat.

In cold water, heat flux from the carapace was the same as from the plastron in the 16 kg turtle which suggests the heat flux may be homogenous over the body surface, *A*_B,_ if the skin is not perfused. This would be expected if there is an even layer of insulation over *A*_B_. Assuming the measured thermal gradient in the larger animal was a true representation of the thermal gradient directly across the plastron, the thermal conductivity, *k*, of leatherback shell can be estimated. The plastron of juvenile leatherbacks of similar mass to those used in these experiments is around 0.02 m thick (personal observations), so *k* is between 0.2–0.3 J s^−1^ K^−1^, which is similar to the thermal conductivity recorded for whale blubber [17].

As the leatherbacks were in steady state at each *T*_W_ and held a stable *T*_B_, the total rate of heat production must equal the total rate of heat loss. Therefore the sum of the heat lost from the plastron, carapace and flippers (ie. *q*_T_) will allow prediction of total heat produced (Table 1 and 2). A caveat is that heat loss is modelled to occur evenly over the entire surface. Total heat production was greatest when the animals were most active and therefore in the coldest water. Despite activity falling as water was warmed from 19 to 25°C, heat loss was constant in both animals and this was probably due to the Q_10_ effect on basal metabolic processes as *T*_B_ was increasing. In 28 and 31°C *T*_W_ the 37 kg leatherbacks heat production rate further increased despite having a very low activity rate, again due to temperature effects on metabolism. In adult leatherbacks that maintain stable *T*_B_'s the heat production rate would be expected to more closely reflect rate of activity because basal metabolic rate should be constant.

### 2. Effect of body mass on thermal gradients

At steady state *T*_B_ – *T*_W_, the total rate of heat transfer, *q*_T_, to the environment equals the rate of heat gained. In leatherbacks, heat is produced endogenously and therefore heat production must approximate the metabolic rate of the animal. Given that resting metabolic rate scales with body mass, *M*_B_ (kg), to the 0.83 power in leatherback sea turtles [18] and rate of heat transfer across an insulation layer is given by Eq. 3 then:
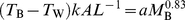
(1)


where *a* is the proportionality coefficient. The body shape of juvenile leatherbacks is similar to those of sub-adults and adults so *A* scales with *M*_B_ to the 2/3 power and *L* will scale with *M_B_* to the 1/3 power since *M*_B_ has dimensions of *L* ^3^. Therefore if *k* is constant then the thermal gradient scales with *M*_B_ to the 0.5 power as:
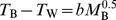
(2)


where *b* is a coefficient that is proportional to the energy expenditure of the animal (ie. doubling *b* corresponds to twice the heat production).

**Figure 3:**
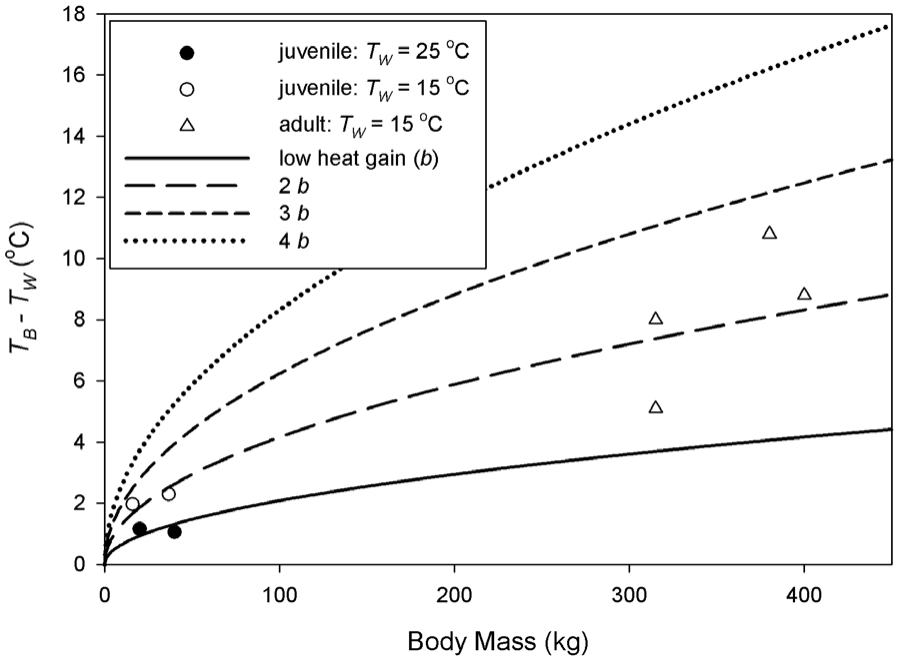
Predicting the thermal gradient adult leatherbacks could maintain. The affect of mass and heat production on the thermal gradient held by leatherbacks were estimated using scaling equations.

The thermal gradients that adult leatherbacks hold can be predicted by scaling the thermal gradient held by juveniles. At 25°C the animals in this study maintained a low level of activity with *T*_B_ – *T*_W_ around 1°C. We fitted these results to Eq. 2 to predict the *T*_B_ – *T*_W_ that animals of different *M*_B_ could maintain with low endogenous heat production and found *b* = 0.21. Figure 3 quantitatively shows the effect that varying heat production, changing *b,* has on the *T*_B_ – *T*_W_ achieved by leatherbacks with minimized heat loss in cold water. The thermal gradient is predicted to be 1.7 and 2.5°C for juveniles of 16 and 37 kg with a heat production rate two times the resting level. This is close to the 2.0 and 2.3°C thermal gradient our animals held when in 16°C tank water when heat loss was double that at 25°C. An average thermal gradient of 8.2°C, measured for adults in 15°C water off of Nova Scotia [7], occurs if heat production is double that of a resting animal.

Metabolic rate of active leatherbacks on a beach was 1.51 W kg^−1^ [8], or five times resting metabolic rate [18]. If leatherbacks maintain such a high heat production rate, our model predicts that a 300 kg animal could hold a maximum *T*_B_ – *T*_W_ gradient of 18.2°C. Therefore leatherbacks swimming in northern temperate waters must maintain substantially elevated metabolic rates to keep *T*_B_ stable. The predictions in Figure 3 assume a thickness of insulation which scales to the 1/3 power with *M*_B_. Animals fatten while foraging so if the insulation layer increases, then for a given heat production rate, a larger thermal gradient will be maintained. James et al. (2005) found leatherbacks off Nova Scotia, Canada to have a 33% greater mass than nesting animals of the same carapace length [19]. If a substantial portion of this increased mass is sub-cutaneous fat insulation will be improved.

Due to scaling effects, the achievable thermal gradient is predicted to scale with *M*_B_, at a given metabolic cost, to the 0.5 power (Eq. 2). Therefore if leatherbacks maintain their *T*_B_ above a minimum value only larger animals should be found foraging in colder waters. Eckert (2002) noted that animals <100 cm carapace length were not observed in *T*_W_ <26°C [13]. James et al. (2006) recorded a leatherback of 148 cm curved carapace length (around 500 kg) repeatedly diving into water of 0.4°C [5]. The model predicts that a 500 kg leatherback would have to have a metabolic rate twice that of our swimming juveniles (four times resting) to keep a body temperature of 20°C in these extremely cold waters.

In warm water our leatherbacks had a similar heat loss rate (Table 1 and 2) from the flippers as the plastron suggesting similar heat flux over their entire body surface when dumping heat in warm water. Presumably this was due to maximizing perfusion of the skin. When coupled with a minimization in flipper stroke frequency the 37 kg leatherback had a *T*_B_ *– T*_W_ of only 0.5°C in the warmest water (31°C). The 16 kg leatherback although having a similar flipper stroke frequency as the 37 kg animal did not increase heat loss as water temperatures rose so its thermal gradient was greater (*T*_B_ – *T*_W_ = 0.8°C). Since surface area scales with *M*_B_ to the 2/3 power and heat production scales to the 0.83, there is very little added heat production per surface area as an animal grows. Therefore, in the tropics, if an adult leatherback routed blood to its entire surface the potential for heat loss should be great enough that the animals will not be in danger of overheating, even when swimming. In the tropics, adult females had a *T*_B_ 1 – 4°C greater than ambient water [6] and *T*_B_ was correlated with *T*_W,_ signifying high rates of heat loss in large leatherbacks.

### 3. Other sea turtles

Sea turtles other than leatherbacks have never been shown to sustain *T*_B_ – *T*_W_s that are large enough to allow migration to cold temperate waters. Free swimming adult loggerhead turtles, for example, generally hold thermal gradients between stomach and water of only 1–2°C [20]. A loggerhead's *T*_B_ thus closely reflects *T*_W,_ and in fact in the Western Atlantic, loggerheads rely on warm waters of the Gulf Stream to overwinter [21]. Kemp's ridleys and green turtles cease to feed and become semi-dormant in water of 15°C [22], whereas adult leatherbacks have been recorded actively feeding in waters as low as 0.4°C [5]. Hard shelled turtles are distributed throughout tropical and subtropical waters again suggesting an inability to maintain a homeostatic *T*_B_ across a wide range of *T*_W_s. A lack of insulation and/or insufficient heat production must underlie the low *T*_B_ – *T*_W_s held by these turtles.

Green turtles do seem to be capable of substantial heat production, at least in the short term, and can maintain pectoral muscles 8°C above *T*_W_ while actively swimming [23]. However, activity in green turtles is unpredictable and when a juvenile was exposed to water at 20°C it remain inactive for 30 minutes before starting swimming [24]. Green turtles of 7–11 kg decrease activity when *T*_W_ is below 20°C and are quiescent in water of 15°C [22]. Juvenile leatherbacks, on the other hand, increase activity as water temperature decreases to at least 16°C and at each *T*_W_ activity levels were constant. In a similar study to ours juvenile green turtles appear to use a thermal strategy much like leatherbacks and accomplish similar *T*_B_ – *T*_W_s [24]. Juvenile greens between 2 and 60 kg held a *T*_B_ – *T*_W_ of 2.2°C in 20°C water and a gradient of 1.7°C in 30°C. The greens had a higher thermal admittance in warm water and were more active in cooler water (20°C) but, as shown above, activity declines below 20°C. Despite superficial similarity between the green turtle data and that presented in this study, in reality, the green turtle results yield little insight into why leatherbacks are capable of traveling to sub-polar waters and greens are not because they did not expose their animals to low temperatures [24]. Due to constant swimming leatherbacks have a more reliable source of endogenous heat which is fuelled by their oceanic, pelagic lifestyle.

To maintain elevated thermal gradients retaining body heat is as important as producing it. Although the pectoral muscles of an actively swimming green sea turtle can be 8°C above *T*_W_ the rest of the body is only 1–2°C above *T*_W_ [23]. This suggests a lack of suitable insulation to maintain large *T*_B_ – *T*_W_s. The measurement of similar internal and external carapace temperatures in a green sea turtle being exposed to intense solar radiation confirms that their carapace is a very poor insulator [24]. Leatherbacks are able to hold a larger gradient than other sea turtles by a combination of large size, a more efficient insulative layer and better control of heat production. Controlling heat loss and gain concurrently is a thermal strategy that allows leatherbacks to exploit the rich foraging grounds of sub-polar waters while avoiding overheating while actively swimming in tropical reproductive zones.

## Materials and Methods

### 1. Animals and Husbandry

#### Ethics Statement

These animals were held for research purposes and all animal care/research standards of the Canadian Council for Animal Care (CCAC) and the UBC Animal Care Committee (UBC Animal Care Protocol: A04-0323) were met.

Leatherback hatchlings were transported from nesting beaches on the British Virgin Islands to the Animal Care Centre, University of British Columbia, Vancouver, B.C., Canada, and raised for a two year period. The experiments reported here were performed on two animals, one weighed 16.1 kg at the start of the experiment and the other weighed 36.7 kg. Both animals were raised in *T*_W_ = 25°C. Throughout the experiment the animals were held in a cylindrical holding tank 2 m in diameter and 1.5 m deep, filled with seawater supplied from the Vancouver Aquarium and Marine Sciences Centre (Vancouver, B.C., Canada). Leatherback turtles were obtained on Canada CITES Import permit CA05CWIM0039 and British Virgin Islands CITES Export certificate CFD062005.

**Figure 4:**
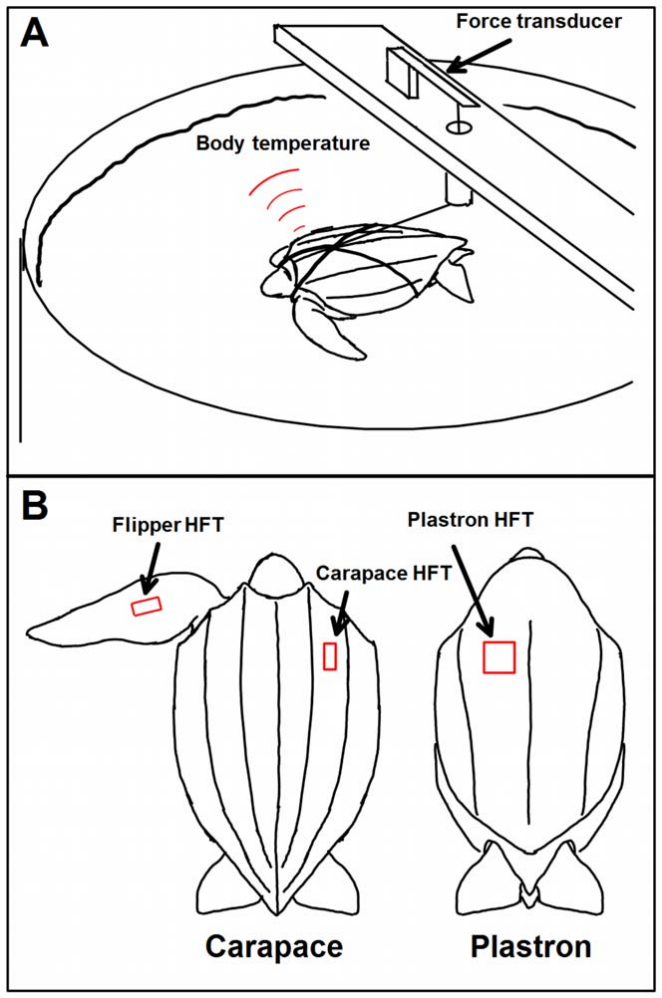
Experimental setup. (A) An illustration of the turtles harnessed in their tanks. (B) The placement of the heat flux transducers (HFT) on the animals.

Leatherbacks have an oceanic-pelagic lifestyle and do not recognize barriers. Consequently, the animals were tethered to the centre of their housing tank by a short length of monofilament fishing line attached to a custom made harness (Figure 4A). The animals could swim or dive without touching the walls or bottom of the tank. The animals were exposed to a 12 hour light/dark cycle. Water quality was maintained by a biological filter, UV sterilization and a protein skimmer. A reservoir tank of equal volume was plumbed into the holding tank. Water temperature of the reservoir was varied and mixing the water in the two tanks allowed *T*_W_ to be changed rapidly. *T*_W_ was maintained within ±0.25°C in the holding tank by a thermostat that controlled hot or cold water flow through a stainless steel heat exchanger. The animal was instrumented and put in the tank at the beginning of the experiment and disturbed only to repair instruments or for feeding which was attempted twice a day.

### 2. Temperature regime

*T*_W_ was changed in a stepwise manner by 3°C increments or decrements starting from 25°C. Water was cooled to 16°C and then warmed from 16 to 31°C and cooled back to 25°C over several days (Figure 1). The lowest maintainable *T_W_* was 16°C due to limitations in the experimental setup and equipment. *T*_W_ was changed by mixing the water in the holding tank with the reservoir tank and the change was completed within 20 minutes of commencing the mix. The water was maintained at each temperature for 11–22 hours for the 37 kg turtle and for 7–24 hours for the 16 kg turtle, long enough for *T*_B_ to become stable (i.e. *T*_B_ change <0.1°C hour^−1^). In total, the experiment with the 16 kg leatherback took 6 days and the experiment with the 37 kg leatherback took 8 days.

### 3. Instrumentation

#### 3.1. *T*_B_ and *T*_W_ recording

A thermocouple (90104, Mon-a-therm® General Purpose, Mallinckrodt Medical, St. Louis, MO, USA) was mounted 5 cm into the housing tank and connected to an electronic thermometer (Physitemp BAT – 12, Sensortek Inc., Clifton, NJ, USA). *T*_W_ was recorded every second. At the beginning of the experiment each animal was given a thermometer pill that was 2.5 cm in length and 1.0 cm in diameter (HT150036, CorTemp™ Equine EXSM Temperature Sensor, HQ Inc., Palmetto, FL, USA). The signal from the pill was picked up by a receiver (HT150001, CorTemp™ Data Recorder, HQ Inc.) housed in a waterproof container suspended 15 cm above the center of the tank. *T*_B_ was recorded every 10 seconds. The thermometer pill had a lifespan of around 3 days after which we briefly removed the turtle from the water and gave the animal a new pill. The animals passed the pills in 1–2 weeks. The electronic thermometer and thermometer pill were calibrated against a mercury thermometer (14-985B, FISHERbrand, Fisher Scientific Ltd., Nepean, ON, Canada).

#### 3.2. Heat flux recording

Heat flux, *Q* (W m^2^), was recorded with heat flux transducers (HFT's; Thermonetics Corp., La Jolla, CA, USA). HFT's are rectangular flat pads that produce a voltage directly proportional to the heat flux through the pad. We attached the transducers to the animals with a thin layer of cyanoacrylate glue. A 3.9×1.9 cm HFT was attached 1/3 of the way along the left front-flipper at the point of largest flipper width (see Figure 4B for placement of HFT's). A 5.7×5.7 cm HFT was attached to the plastron 10 cm distally from the anterior edge and 2.5 cm to the right of each animal's center line. A 3.9×1.9 cm HFT was attached only to the carapace of the 16 kg turtle. The HFT wires ran from the turtle, along the bottom of the tank and then out of the tank to a multiple channel signal conditioner (CyberAmp 320, Axon Instruments) and, after amplification, the signals were analog to digital, A – D, converted and recorded at a rate of 1 Hz. HFT's were attached so that a positive value of heat flux represented heat transferring from the turtle to the water.

#### 3.3. Activity recording

A wooden plank with a short section of 14 mm (internal diameter) PVC pipe inserted through it was placed across the tank. A length of monofilament fishing line attached to the animal's harness (Figure 1A) passed through the PVC pipe to a force transducer (FT03C, Grass Instrument Co., Quincy, MA, USA). The PVC pipe redirected the force exerted by the turtle vertically so that recorded force was unaffected by the direction in which the animal was swimming. The signal from the force transducer was A–D converted and recorded at a rate of 5 Hz. Flipper strokes per minute (SPM) were used as an index of activity.

### 4. Data recording and analysis

**Figure 5:**
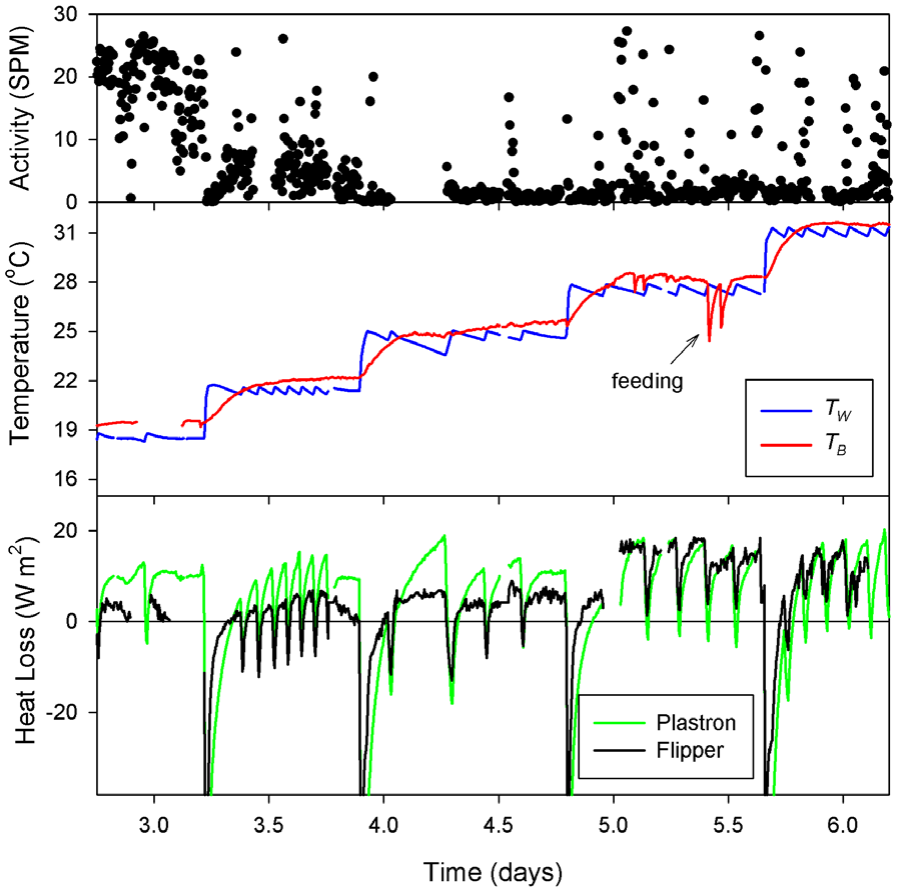
All data was simultaneously recorded. Activity, water and body temperature and heat fluxes recorded simultaneously from the 37 kg leatherback during stepwise increases in water temperature.

All analog signals were digitized with an analog to digital (A–D) converter (USB – 1208LS, Measurement Computing, Norton, MA, USA) and recorded on a notebook computer with TracerDAQ® (Measurement Computing). All feeding events and data collected when an animal had been disturbed due to re-instrumentation were discarded. Heat flux data was calibrated using calibration curves provided by the manufacturer. Acq*Knowledge*® software (version 3.7.5, BIOPAC Systems Inc., Santa Barbara, CA, USA) was used to detect peaks in the data from the force transducer. Each peak corresponded to a flipper stroke. This force data was then converted to strokes per minute (SPM) and averaged over the period the animal was held at a given temperature. All other data were taken when the animal was close to steady state which was considered to be when *T*_B_ was changing at a rate <0.1°C hour^−1^. Therefore, in the 16 kg animal no data was included that was <6 hours after the water change, and in the 37 kg animal no data <7.5 hours was included. Water temperature in the holding tank fluctuated by ±0.25°C and all heat fluxes as well as the thermal gradient fluctuated in time with *T*_W_ oscillations (Figure 5). To get the steady state heat flux and thermal gradient maintained for a given *T*_W_ we averaged that small section of the data when *T*_W_ was ±0 0.075°C around the mean value for that trial. The actual period averaged was usually about 30 minutes.

### 5. Calculations

#### 5.1. Surface area

Surface area of both leatherbacks was measured post-mortem. When the large and small animals died of natural causes their masses were 42.0 and 22.3 kg, respectively. Half the carapace, half the plastron, the ventral side of the left front and rear flipper of each animal was covered in paper. The paper was then removed, laid flat and the area was determined by creating geometric shapes with an easily measured area. Total body area (*A*_B_) was twice the measured area of plastron and carapace and total flipper area (*A*_F_) was four times the measured area of both front and rear flippers. These areas were scaled allometrically with *M_B_* in order to predict the surface area of the turtles during experimentation.

#### 5.2. Total heat transfer rate

The total rate of heat transfer, *q*_T_ (W), from the turtle to the tank was estimated as *A*_B_ *Q*_P_+*A*_F_ *Q*_F_ where *Q*_P_ and *Q*_F_ are heat flux (W m^2^) from the plastron and flipper, respectively. The fraction of *q*_T_ that was lost through the flippers is *A*_F_ *Q*_F_/*q*_T_. The remaining heat loss was from the body.

#### 5.3. Thermal admittance of the plastron

The rate of heat transfer (W) across an insulation layer of thermal conductivity *k* (W m^−1^ K^−1^) and thickness *L* (m) is given by:
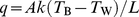
(3)


where *T*_B_ – *T*_W_ is the thermal gradient from one side of the layer of insulation to the other, and *A* (m^2^) is the area over which heat is lost. To give an index of the control of heat loss through the plastron exhibited by the turtles we divided measured *Q*_P_ from the HFT (ie., *q*/*A*) by the measured *T*_B_ – *T*_W_. This gives a rate at which heat energy transfers across a given insulator of area 1 m^2^ driven by a thermal gradient of 1°C and is referred to as the thermal admittance (W m^−2^°C^−1^). Thermal admittance is also equal to *k*/*L*.
